# Mitochondrial Gene Regulation and Pain Susceptibility: A Multi-Omics Causal Inference Study

**DOI:** 10.3390/ijms26178690

**Published:** 2025-09-06

**Authors:** Chien-Cheng Liu

**Affiliations:** 1 Department of Anesthesiology, E-Da Hospital, I-Shou University, Kaohsiung City 82445, Taiwan; jasperliu1552@isu.edu.tw or jasperliu1552@gmail.com; 2 School of Medicine, I-Shou University, Kaohsiung City 82445, Taiwan; 3 Department of Nursing, College of Medicine, I-Shou University, Kaohsiung City 82445, Taiwan; 4 Department of Data Science and Analytics, College of Intelligent Science and Technology, I-Shou University, Kaohsiung City 82445, Taiwan; 5 Interventional Pain Management Center, E-Da Cancer Hospital, Kaohsiung City 82445, Taiwan

**Keywords:** neuralgia, neuritis, Mendelian randomization, mitochondria, methylation, gene expression, protein

## Abstract

The causal contributions of specific mitochondrial genes to common pain phenotypes remain unclear. We employed a multi-omics Mendelian randomization (SMR) approach, integrating QTL data (expression, methylation, protein) for mitochondrial genes with GWAS summary statistics for seven pain phenotypes. We identified 18 candidate genes with robust SMR associations across omics layers. However, strong colocalization evidence (PP.H4 > 0.7) was largely absent, pointing towards complex genetic architectures. A notable exception was a strong signal for a shared causal variant found at the methylation level for the MCL1 gene in hip pain (PP.H4 = 0.962), nominating it as a high-confidence candidate. Additionally, genetically predicted higher protein levels of Glycine amidinotransferase (GATM) showed consistent protective associations with neck or shoulder, back, and knee pain. This study provides novel evidence for mitochondrial gene regulation in pain, highlighting the GATM pathway as protective and identifying MCL1 methylation as a potential causal mechanism in hip pain.

## 1. Introduction

Chronic pain, including neuralgia and neuritis, is a major global health challenge with a complex pathophysiology. Among the factors contributing to this complexity, emerging evidence highlights the critical role of cellular bioenergetics and mitochondrial function in neuronal health and sensory processing, as mitochondria are essential for neuronal energy supply, calcium homeostasis, and redox signaling vital for normal nociception [1,2].

Mitochondrial dysfunction is increasingly implicated in specific pain conditions, such as neuropathic pain associated with nuclear polymerase gamma (POLG) mutations or certain mitochondrial DNA (mtDNA) variants [3]. However, systematic investigation into the broader mitochondrial gene repertoire’s contribution to common neuralgia and neuritis phenotypes remains limited, particularly through causal inference methods. To address this knowledge gap, this study leverages a multi-omics summary-data-based Mendelian randomization (SMR) approach [4,5]. By integrating large-scale genetic data for methylation (mQTL), expression (eQTL), and protein quantitative trait loci (pQTL) with genome-wide association study (GWAS) summary statistics [6] for seven distinct neuralgia and neuritis phenotypes, we aim to systematically identify potential causal links between mitochondrial gene regulation and susceptibility to these debilitating pain conditions.

## 2. Results

### 2.1. Integrated Multi-Omics SMR Identifies Candidate Mitochondrial Genes Associated with Pain Phenotypes

**Figure 1:**
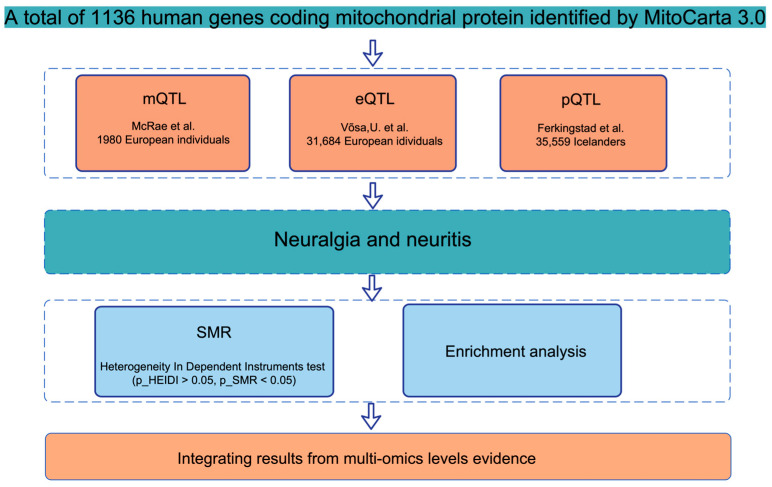
The analytical workflow. The analysis was based on 1136 human genes coding for mitochondrial proteins identified from MitoCarta 3.0 [7]. The workflow integrated multi-omics data, including DNA methylation quantitative trait loci (mQTL) from McRae et al. [8], gene expression quantitative trait loci (eQTL) from Võsa, U. et al. [9], and protein quantitative trait loci (pQTL) from Ferkingstad et al. [10]. SMR, summary-data-based Mendelian randomization; HEIDI, heterogeneity in dependent instruments.

Following the analytical workflow (Figure 1), we performed a multi-omics SMR analysis to identify mitochondrial genes associated with seven neuralgia and neuritis phenotypes using UK Biobank GWAS summary statistics (Supplementary Table S2). We systematically evaluated genetic instruments for methylation (mQTL), expression (eQTL), and protein (pQTL) across 1136 mitochondrial genes from the MitoCarta 3.0 database (Supplementary Table S1).

After applying significance and heterogeneity filters (*p*_SMR < 0.05 and *p*_HEIDI > 0.05), our integrative analysis yielded 18 unique mitochondrial genes robustly associated with at least one pain outcome across multiple omics layers. The specific genes identified for each pain phenotype are detailed in Table 1. Notably, the gene GATM was associated with three distinct conditions: neck or shoulder pain, back pain, and knee pain.

Subsequent colocalization analyses were performed to assess whether these SMR associations were driven by shared causal variants. While strong evidence for colocalization was largely absent, we identified a notable exception: the association between a methylation QTL (mQTL) for the MCL1 gene and hip pain demonstrated strong evidence for a shared causal variant (PP.H4 = 0.962), as noted in Table 1. For the remaining associations with low PP.H4, we performed a deeper analysis of the full posterior probability distributions to investigate alternative genetic scenarios, such as linkage disequilibrium. The complete results of these expanded analyses are presented in Supplementary Table S4.

### 2.2. Effect Sizes and Directionality Reveal Diverse and Complex Roles of Mitochondrial Genes

**Figure 2:**
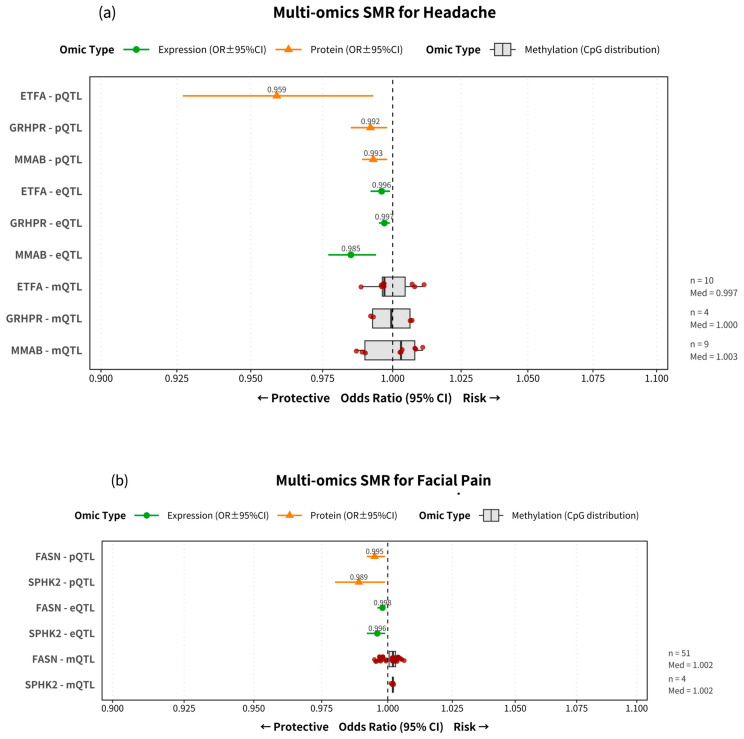

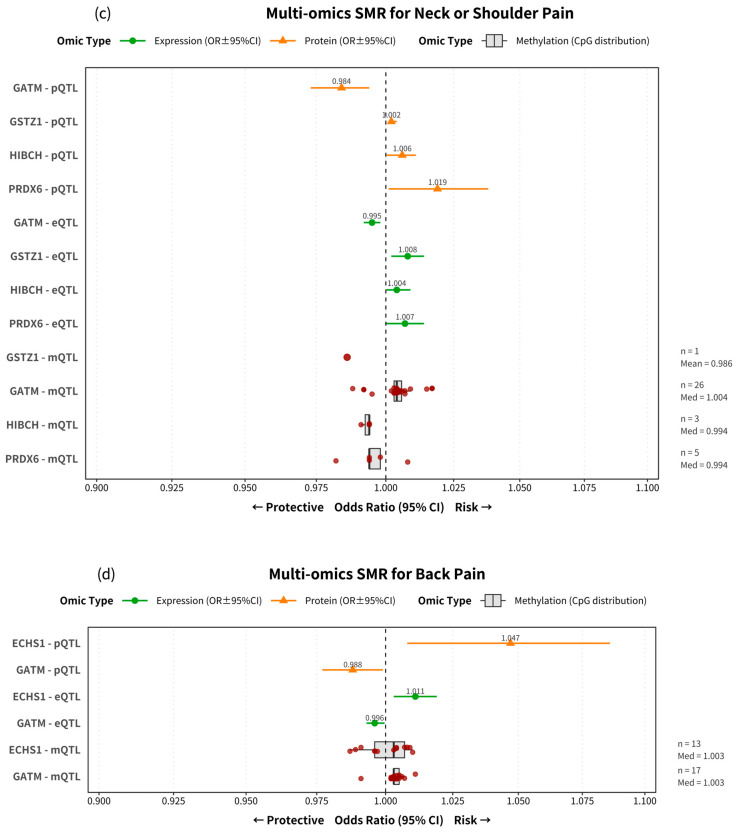

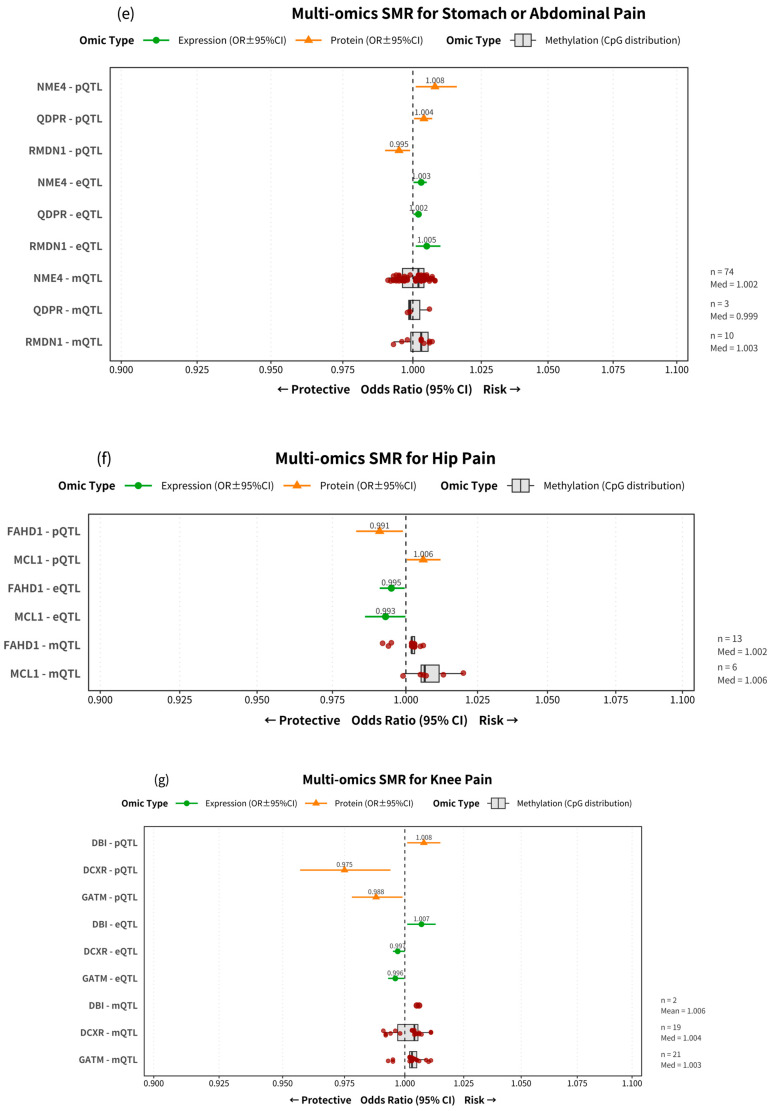
Multi-omics SMR associations for neuralgia phenotypes. Forest plots illustrating the causal association estimates between mitochondrial gene regulation and seven pain phenotypes. The figure comprises seven panels, each displaying the results for a specific phenotype: (**a**) headache, (**b**) facial pain, (**c**) neck or shoulder pain, (**d**) back pain, (**e**) stomach or abdominal pain, (**f**) hip pain, and (**g**) knee pain. Within each panel, the most significant genes identified via SMR are displayed. Odds Ratios (ORs) and their 95% Confidence Intervals (CIs) are derived from summary-data-based Mendelian randomization (SMR) analysis. The estimated OR and 95% CI for each protein (pQTL) and expression (eQTL) are represented by a point and a horizontal line. For methylation (mQTL), the results are summarized based on the number of significant CpG probes (*n*), which is annotated on the right side of the plot. For genes with three or more significant probes (*n* ≥ 3), the distribution of ORs is shown as a box plot, where the central line represents the median (Med) effect, accompanied by individual red dots for each probe. For genes with fewer than three probes (*n* < 3), only the individual points are displayed, and their summary effect is represented by the mean (Mean). The vertical dashed line indicates the null effect (OR = 1.0). ORs below 1.0 suggest a potential protective effect, while ORs above 1.0 suggest a potential risk-increasing effect.

We next examined the directionality and consistency of the 18 candidate gene associations across the different omic layers. The multi-omics SMR effect estimates for each pain phenotype are summarized in Figure 2. The complete forest plots for each phenotype, detailing all individual mQTL associations, are provided in Supplementary Figure S1a–g. Our analysis of these results revealed three distinct patterns: consistent protective effects, consistent risk-increasing effects, and complex or conflicting effects between molecular layers.

First, several genes showed consistent protective associations, where genetically predicted higher expression and protein levels were linked to a reduced risk of pain. The most prominent example was GATM, which demonstrated a robust protective effect against neck or shoulder pain (pQTL OR = 0.984), back pain (pQTL OR = 0.988), and knee pain (pQTL OR = 0.988) (Figure 2c,d,g). Similarly, genes identified for headache (e.g., ETFA, GRHPR, MMAB) and facial pain (e.g., FASN, SPHK2) also consistently showed protective trends at both the expression and protein levels (Figure 2a,b).

In contrast, other genes were associated with an increased risk of pain. For instance, higher expression and protein levels of ECHS1 were linked to a greater risk for back pain (pQTL OR = 1.047) (Figure 2d). A similar risk-increasing pattern was observed for DBI in knee pain (pQTL OR = 1.008) (Figure 2g), and for GSTZ1, HIBCH, and PRDX6 in neck or shoulder pain (Figure 2c).

Finally, our analysis highlighted the complexity of mitochondrial gene regulation, revealing conflicting signals between different molecular layers for some genes. A clear example is MCL1 in hip pain, where higher expression (eQTL) was protective (OR = 0.993), but higher protein levels (pQTL) suggested increased risk (OR = 1.006) (Figure 2f). A similar conflict was observed for RMDN1 in stomach or abdominal pain (Figure 2e). Furthermore, the effects of DNA methylation (mQTL) often showed significant heterogeneity; the boxplots summarizing CpG site effects frequently spanned the null value (OR = 1) and had medians close to 1.0, even for genes with clear directional effects at the eQTL or pQTL level. This suggests that the regulatory impact of these genes on pain susceptibility is highly context-dependent and varies across different biological layers.

### 2.3. Shared Gene Patterns and Functional Insights from Enrichment Analysis

**Figure 3:**
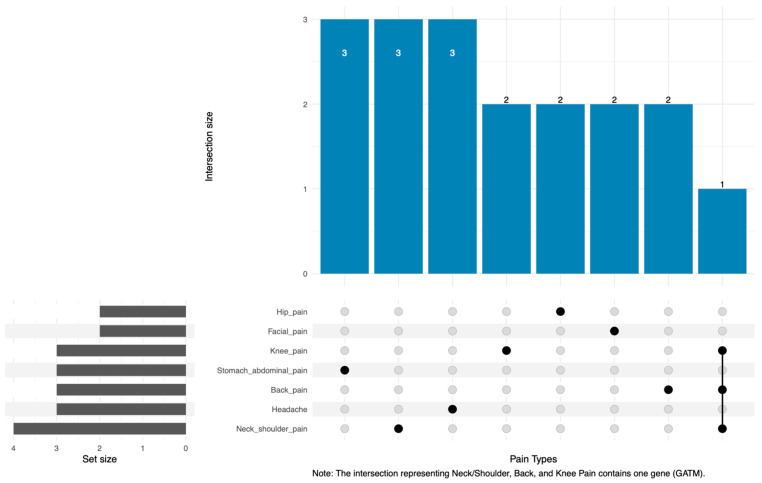
Overlap patterns of integrated mitochondrial genes across seven neuralgia phenotypes. The UpSet plot visualizes the distribution and intersections of the 18 mitochondrial genes identified via integrated multi-omics SMR analysis (listed in Table 1) across the seven pain phenotypes, which represent the ‘sets’. The horizontal bar chart on the left indicates the set size, the total number of integrated genes associated with each respective pain phenotype. The vertical bar chart at the top represents the intersection size, the number of genes belonging to each specific intersection category. The matrix below the top bar chart indicates set membership for each intersection: solid black dots, connected by a solid black line, identify the pain phenotypes included in that specific set, while light gray dots indicate phenotypes that are not part of the set. Notably, the intersection representing genes exclusively associated with neck or shoulder pain, back pain, and knee pain contains only one gene (GATM).

The analysis of 18 integrated mitochondrial genes predominantly revealed phenotype-specific associations, with most genes correlating with a single pain condition (Figure 3). Notably, GATM was the only gene associated with multiple phenotypes; it was concurrently linked to neck or shoulder pain, back pain, and knee pain. GATM may, therefore, serve as a shared mitochondrial node that influences these interconnected musculoskeletal conditions.

To further elucidate the biological functions of the associated genes, we performed gene ontology (GO) enrichment analysis and visualized the results as enrichment network maps. In these networks, GO terms are represented as nodes that are connected if they share overlapping genes, thereby revealing distinct functional clusters. To specifically highlight the contribution of GATM, which was pleiotropically associated with three pain phenotypes, its related GO terms were distinctly visualized as orange diamonds.

**Figure 4:**
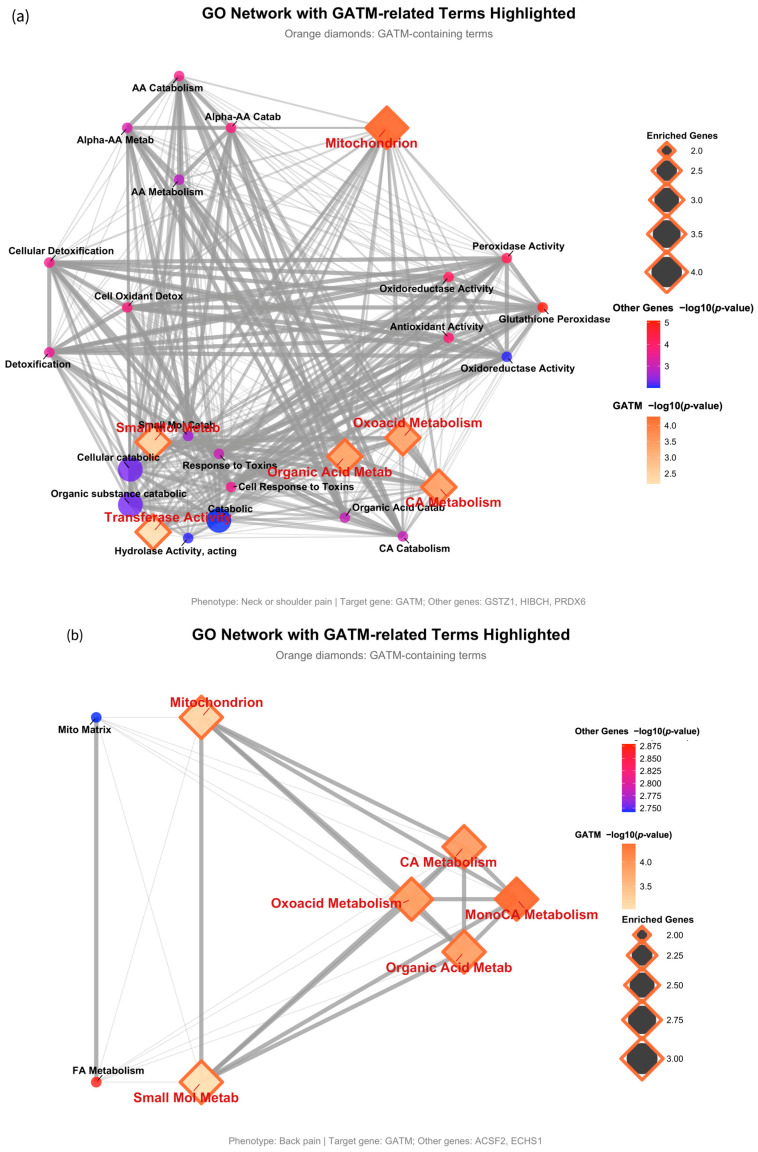

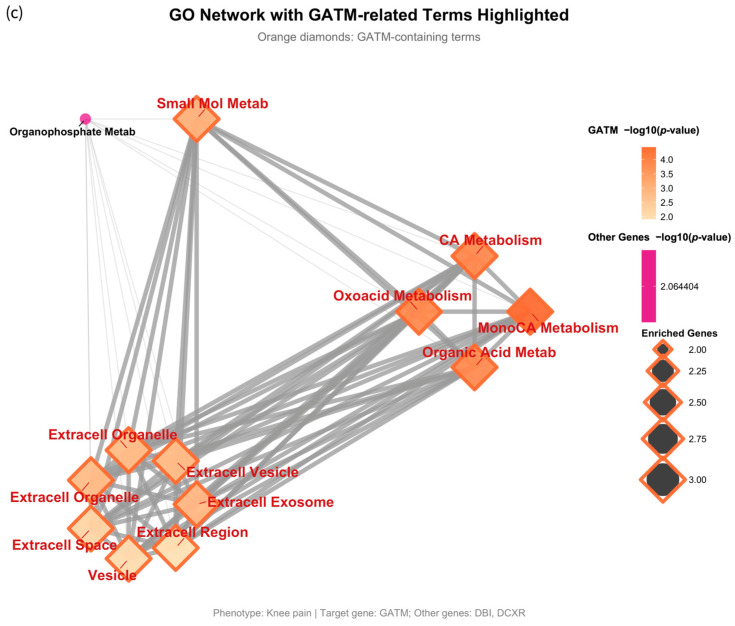
Gene ontology (GO) enrichment network maps for GATM-associated pain phenotypes. To visualize the biological functions of the associated gene sets, gene ontology (GO) enrichment analysis was performed using the clusterProfiler package; significance was determined based on a Benjamini–Hochberg adjusted *p*-value (p.adjust) < 0.05 and a minimum gene count of two per term. The results for (**a**) neck or shoulder pain, (**b**) back pain, and (**c**) knee pain are displayed as enrichment network maps. In these networks, each node represents a significantly enriched GO term, and the edges connect terms that share overlapping genes, grouping them into functional clusters. The thickness of the gray connecting lines (edges) represents the degree of gene overlap between GO terms—thicker lines indicate higher proportions of shared genes between connected terms. The size of each node is proportional to the number of genes enriched in that term (enriched genes). The color intensity reflects the statistical significance of the enrichment, represented as −log10 (*p*-value). To specifically highlight the role of the target gene GATM, nodes representing GO terms that include GATM are displayed as orange diamonds, with their color gradient corresponding to the GATM-specific enrichment *p*-value. Nodes for terms enriched only by other genes in the set are displayed as circles, with their color gradient reflecting their respective *p*-values. The abbreviated GO term labels shown in the plots correspond to the full GO term descriptions available in Supplementary Table S3.

For neck or shoulder pain, the analysis revealed a dense network where GATM acts as a central hub connecting core mitochondrial metabolic processes, such as ‘alpha-amino acid catabolic process’ and ‘oxoacid metabolism’, with a prominent functional cluster related to redox balance and detoxification, including terms like ‘cellular oxidant detoxification’ and ‘glutathione peroxidase activity’ driven by the other associated genes (GSTZ1, HIBCH, PRDX6) (Figure 4a).

Similarly, for back pain, the gene set (GATM, ACSF2, ECHS1) was almost exclusively enriched in a tightly interconnected network of mitochondrial metabolism. GATM bridged various organic and carboxylic acid metabolic pathways, while other genes contributed specifically to ‘fatty acid metabolism’ within the ‘mitochondrial matrix’ (Figure 4b).

Interestingly, the gene set for knee pain (GATM, DBI, DCXR) showed GATM connecting its characteristic metabolic hub with a unique and significant cluster of terms related to the extracellular environment, such as ‘extracellular exosome’ and ‘extracellular vesicle’ (Figure 4c). This suggests a potential role for GATM not only in cellular bioenergetics but also in intercellular communication in the context of knee pain.

Significant GO enrichment was also observed for phenotypes not associated with GATM. The gene set for headache (ETFA, GRHPR, MMAB) was enriched in two primary functional clusters: one centered on core mitochondrial energy pathways, including ‘oxoacid metabolism’; and another related to molecular binding functions, such as ‘nucleoside phosphate binding’ (Supplementary Figure S2a). For facial pain, the associated genes (FASN, SPHK2) showed a highly specific enrichment in a network of ‘lipid biosynthetic’ and ‘lipid metabolic’ processes (Supplementary Figure S2b). In contrast, no statistically significant GO term enrichment (p.adjust < 0.05, count ≥ 2) was found for the gene sets related to stomach or abdominal pain or hip pain. Additionally, no significantly enriched KEGG pathways were identified for any of the evaluated gene sets.

## 3. Discussion

This study employed a multi-omics SMR framework to investigate mitochondrial genes in seven pain phenotypes, identifying 18 candidates with multi-layer regulatory signals. Our analysis offers novel insights into the complex genetic basis of pain, notably by nominating MCL1 methylation as a high-confidence causal candidate in hip pain and highlighting a consistent protective role for GATM across musculoskeletal pain conditions. These findings prioritize specific mitochondrial pathways for further validation.

While growing evidence links mitochondrial dysfunction (involving processes like calcium homeostasis and reactive oxygen species (ROS) management that are relevant to neuroinflammation) to chronic pain pathophysiology [1,2], systematic exploration using advanced genetic causal inference, particularly multi-omics SMR/colocalization across diverse pain phenotypes for the nuclear-encoded mitochondrial gene repertoire, remains limited. Despite known links (e.g., mtDNA variants in chemotherapy-induced peripheral neuropathy, CIPN) [3] and related SMR studies in conditions like T2DM-neuropathy [11], dedicated multi-phenotype pain studies using this comprehensive framework are scarce. Our study addresses this gap using a robust methodology. SMR leverages genetic variants as instruments, mitigating confounding and strengthening causal inference on gene regulation’s role in pain susceptibility over observational methods [4]. Integrating multi-omics data (epigenetic, transcriptomic, proteomic) provides converging evidence, boosting confidence in the functional relevance of candidates like the 18 identified genes [5]. Utilizing large-scale GWAS/QTL data maximized power [6], while HEIDI/colocalization analyses added rigor in assessing shared causal variants versus LD confounding [12]. This multi-layered genetic approach effectively nominates specific mitochondrial genes for mechanistic investigation in pain pathways.

A critical observation, consistent with similar complex trait analyses [13], was that strong evidence for a shared causal variant (PP.H4 > 0.7) was not widespread across the 18 SMR-significant associations. The juxtaposition of significant SMR signals with low colocalization probabilities necessitated a deeper investigation into the underlying genetic architecture of these loci by evaluating the full posterior probability distribution (H0–H4) [14]. This deeper analysis (Supplementary Table S4) yielded our study’s most significant signal: strong evidence for a shared causal variant (PP.H4 = 0.962) between a methylation QTL for the apoptosis regulator MCL1 and hip pain risk. While this signal is compelling, we interpret it with caution, as it was specific to the methylation level and was not observed in our eQTL or pQTL analyses. Therefore, we propose MCL1 as a high-confidence candidate that highlights a potential epigenetic mechanism in hip pain, which warrants further functional validation. For other associations, a prominent alternative was strong evidence for Hypothesis H3 [14], exemplified by the link between an mQTL for MMAB and headache (PP.H3 = 0.994), which indicates the SMR signal is likely genuine but driven by two separate, closely linked variants.

These findings highlight that a lack of colocalization does not negate a gene’s potential causal role but instead points towards a spectrum of complex scenarios. Other possibilities that require consideration include horizontal pleiotropy missed by HEIDI [15], statistical power limitations [16], methodological choices [16,17], or allelic heterogeneity. Thus, our expanded analysis successfully uses the full posterior probability distribution to characterize a landscape of locus-specific genetic architectures, nominating causal genes within complex genomic regions.

Examining association patterns across seven pain phenotypes revealed shared and phenotype-specific genetic links (Figure 3), aligning with expectations of common pathobiological pathways alongside unique characteristics. A striking finding was the recurrent protective association of GATM (Glycine amidinotransferase) across neck or shoulder, back, and knee pain, as indicated by SMR analysis of genetically predicted protein levels. This suggests GATM involvement in generalized mechanisms relevant to common musculoskeletal or neuropathic pain conditions. To address the potential issue of tissue specificity arising from the use of blood-based QTL data, we verified the expression profile of GATM using the Genotype-Tissue Expression (GTEx) portal [18]. The data confirm that GATM is expressed in numerous pain-relevant tissues, including skeletal muscle (median TPM = 15.84), tibial nerve (median TPM = 12.31), spinal cord (cervical c-1) (median TPM = 18.51), and various brain regions. This multi-tissue expression pattern, with transcript-per-million (TPM) values indicating meaningful expression levels, enhances the biological plausibility of GATM’s systemic role in pain modulation, suggesting its function is not confined to the blood and is indeed relevant to the tissues implicated in the pain phenotypes studied. Furthermore, a systematic examination revealed that the majority of our 18 candidate genes are expressed across these pain-relevant tissues (see Supplementary Table S5).

Mechanistically, GATM is the rate-limiting enzyme in the biosynthesis of creatine, a molecule vital for energy homeostasis in high-demand tissues like muscle and neurons [19]. The observed protective association, therefore, likely stems from the multi-faceted benefits of a robust creatine system. These include enhanced bioenergetics that improve tissue resilience against metabolic stress [20], potent anti-inflammatory and antioxidant effects that can mitigate pain-related inflammation [21], and direct neuroprotective functions that support neuronal health and may modulate pain signaling pathways [22,23].

In contrast to GATM’s broad associations, our findings for other genes suggest phenotype-specific links to pathways pertinent to unique pain syndrome contexts. This underscores the potential heterogeneity in mitochondrial contributions across pain conditions. Our investigation has now identified novel mechanistic hypotheses for the associations between MCL1 and hip pain, and MMAB and headache, suggesting distinct ways mitochondrial dysfunction can modulate pain [24]. The role of MCL1, a key regulator of apoptosis, appears linked to inflammation and cellular survival within joint tissues [25]. Its established involvement in the chronic inflammation of rheumatoid arthritis and its protective function in chondrocytes in osteoarthritis provide direct mechanisms for its association with hip pain [26]. In contrast, the MMAB gene’s link to headache stems from metabolic dysfunction [27]. Deficiency in MMAB, which is critical for vitamin B12 metabolism, leads to the accumulation of neurotoxic methylmalonic acid (MMA) [28]. Elevated MMA impairs neuronal energy metabolism by inhibiting mitochondrial respiratory chain function, a plausible trigger for headaches and migraines [29].

While the primary pathways are distinct—MCL1 in inflammation-driven cell survival and MMAB in metabolic neurotoxicity—both may ultimately converge on downstream effectors like oxidative stress, a known driver of chronic pain [30]. These findings highlight the diverse roles of mitochondrial genes in the pathogenesis of specific pain phenotypes and identify them as potential therapeutic targets [31]. We have also compiled a summary of their core functions and their potential relevance to pain pathophysiology in Supplementary Table S6 [32,33,34,35,36,37,38,39,40,41,42,43,44,45,46]. This resource may help in forming further hypotheses for future research.

While leveraging multi-omics SMR strengths for hypothesis generation, our study has several limitations that require careful consideration. First, the validity of MR/SMR relies on instrumental variable assumptions that are hard to fully verify [47], and residual confounding from complex horizontal pleiotropy cannot be ruled out, even with the use of HEIDI and colocalization analyses [15,48].

Second, a significant and valid limitation of our study is the use of QTL data derived primarily from blood, which may not fully represent the gene regulatory mechanisms in the most causally relevant tissues for pain (e.g., dorsal root ganglia, spinal cord, muscle) [49]. Critically for pain research, tissue specificity remains a major challenge, as QTLs from accessible tissues like blood may not reflect regulation in relevant pain-related tissues [50]. This mismatch could lead to biased estimates or false negatives. To partially mitigate this, we examined the expression of our candidate genes in the GTEx database and confirmed that most, including our primary candidate GATM, are expressed in skeletal muscle and nervous tissues (Supplementary Table S5). This lends support to their potential roles in the identified pain phenotypes. Nevertheless, this tissue mismatch may also explain the general lack of strong colocalization in our study, as a true causal link is unlikely to colocalize with blood QTLs if the mechanism operates tissue-specifically [51].

Finally, we acknowledge several key statistical and methodological considerations. A primary consideration is multiple testing; in this exploratory, hypothesis-generating study, we did not apply a formal study-wide correction such as FDR for the final selection of our 18 integrated candidates. Instead, we prioritized a strategy of cross-omics validation. The statistical probability of a single gene emerging as a false positive by chance at *p* < 0.05 across three independent molecular datasets is exceedingly low (*p* < 0.05^3^ or 0.000125). This convergence of evidence acts as a stringent filter that substantively mitigates the risk of false positives. Further limitations include potential statistical power constraints for multi-omics integration and colocalization [16], which may obscure some true associations, and the influence of methodological choices (e.g., window size, priors, single-variant assumption) [17]. Taking these factors into account, our list of candidates should be interpreted as high-priority hypotheses requiring further validation, rather than as definitively confirmed causal genes.

## 4. Materials and Methods

### 4.1. Comprehensive Study Design and Data Integration

This study employed a multi-omics Mendelian randomization (MR) framework, primarily utilizing summary-data-based MR (SMR), to delineate potential causal associations between mitochondrial genes and seven distinct neuralgia and neuritis phenotypes by assessing the causal influences of mitochondrial expression (eQTL), methylation (mQTL), and protein levels (pQTL) on these outcomes. Subsequent colocalization analyses examined the congruence of genetic signals between mitochondrial quantitative trait loci (QTLs) and these phenotypes. Gene set enrichment analyses probed the biological ramifications of identified mitochondrial genes. The integrated analytical strategy, covering gene selection, causal inference, and functional annotation, is depicted in Figure 1.

### 4.2. Acquisition and Processing of mQTL, eQTL, and pQTL Data

To identify genetic variants that affect mitochondrial function, we used a curated list of 1136 mitochondria-related genes from MitoCarta3.0 [7], which is a thoroughly updated resource that includes information on gene localization and pathways (see Supplementary Table S1). We then identified and processed cis-acting single nucleotide polymorphism (SNPs) (within ±1000 kb of each target gene) across three molecular layers.

#### 4.2.1. DNA Methylation Quantitative Trait Loci (mQTL)

mQTL data were obtained from meta-analyses of the Brisbane Systems Genetics Study (*n* = 614) and Lothian Birth Cohorts (*n* = 1366), comprising a total of 1980 European individuals [8]. The intensities of methylation probes were normalized using generalized linear modeling (logistic link), accounting for technical (microarray chip), demographic (age, sex), and interaction factors (age^2^, sex × age, sex × age^2^) [8]. After normalization, linkage disequilibrium (LD) pruning was conducted using PLINK software (version 2.0) (parameters: clump_kb = 10,000, clump_r^2^ = 0.001) with the 1000 Genomes EUR reference panel [52,53]. Cis-mQTLs that achieved genome-wide significance (*p* < 5 × 10^−8^) were selected for SMR analysis.

#### 4.2.2. Gene Expression Quantitative Trait Loci (eQTL)

Summary-level eQTL statistics were obtained from the eQTLGen Consortium (https://www.eqtlgen.org), which includes peripheral blood samples from 31,684 European individuals [9]. Gene expression measurements derived from RNA-sequencing platforms underwent trimmed mean of M-values (TMM) normalization followed by log2-transformation, whereas expression data originating from microarray platforms were subjected to quantile normalization. Linear regression models were employed to adjust for technical covariates (e.g., batch effects), demographic factors (age and sex), and principal components (PCs). Cis-eQTL SNPs linked to mitochondrial genes that reached genome-wide significance (*p* < 5 × 10^−8^) were retained for further analysis.

#### 4.2.3. Protein Quantitative Trait Loci (pQTL)

pQTL data were sourced from the DECODE study’s plasma proteomics assays (*n* = 35,559 Icelanders) [10]. Protein abundance was normalized using rank-based inverse normal transformation (RINT). Linear regression models adjusted for technical covariates (processing time, batch) and demographics (age, sex). Genetic variants significantly associated (*p* < 1.8 × 10^−9^) with mitochondria-related proteins were selected for further analysis.

Using the MitoCarta3.0 gene list as a reference, this process resulted in the identification of 704 methylation-associated, 910 expression-associated, and 109 protein-associated mitochondrial genes from the respective mQTL, eQTL, and pQTL datasets for further investigation.

### 4.3. Acquisition of Outcome GWAS Data and Quality Control of Instrumental Variables

Summary-level genetic association statistics pertaining to seven phenotypes associated with neuralgia were obtained from the MRC-IEU OpenGWAS database (https://gwas.mrcieu.ac.uk), which is derived from the UK Biobank cohort comprising 461,857 individuals of European ancestry [54]. The specific phenotypes, along with their corresponding GWAS identifiers and case/control counts, are as follows: headache (UKB-B-12181; 93,308 cases/368,549 controls), facial pain (UKB-B-17107; 8595 cases/453,262 controls), neck or shoulder pain (UKB-B-18596; 106,521 cases/355,336 controls), back pain (UKB-B-9838; 118,471 cases/343,386 controls), stomach or abdominal pain (UKB-B-11413; 39,646 cases/422,211 controls), hip pain (UKB-B-7289; 52,087 cases/409,770 controls), and knee pain (UKB-B-16254; 98,704 cases/363,153 controls) (further details can be found in Supplementary Table S2). It is noteworthy that there was no overlap in samples between the exposure (QTL) and outcome (GWAS) datasets.

Prior to conducting the SMR analysis, instrumental variables (SNPs) derived from the QTL datasets underwent a comprehensive quality control process, which included the following steps:

- Cis-region Selection: SNPs located within ±1000 kb of the target gene were considered.
- Significance Threshold: A significance level of *p* < 5 × 10^−8^ was required for m/eQTL analyses, while a threshold of *p* < 1.8 × 10^−9^ was set for pQTL analyses.
- LD Pruning: SNPs exhibiting strong linkage disequilibrium (r^2^ > 0.9, based on the 1000 Genomes EUR dataset) were excluded.
- Weak Instrument Filtering: SNPs with an F-statistic of less than 10 were removed from consideration.
- Allele Frequency Concordance: SNPs demonstrating frequency discrepancies greater than 0.2 between the LD reference, QTL, and GWAS datasets were filtered out.
- Harmonization: A meticulous alignment of effect alleles and sizes between the exposure and outcome datasets was performed to ensure consistent estimation in the SMR analysis.

### 4.4. Statistical Analysis and Causal Inference Through Summary-Data-Based Mendelian Randomization (SMR)

Summary-data-based Mendelian randomization (SMR) was employed to assess potential causal relationships between molecular exposures, including mitochondrial gene methylation, expression, and protein levels, and specific neuralgia phenotypes [55]. Independent genetic variants, specifically single nucleotide polymorphisms (SNPs) that met quality control criteria, were utilized as instrumental variables (IVs). These IVs were presumed to have a strong association with the exposures (e.g., gene expression) while remaining independent of the outcomes (disease phenotype), conditional on the exposure and confounding variables. The causal effect in SMR (β_Exposure→Outcome_ = β_SNP→Outcome_/β_SNP→Exposure_) was estimated, and significant associations were identified through a multi-stage filtering strategy. First, for initial screening within each of the three omics layers (mQTL, eQTL, pQTL), we identified associations that passed a nominal significance threshold (*p*_SMR < 0.05). To address potential confounding from linkage disequilibrium, these associations were then filtered using the heterogeneity in dependent instruments (HEIDI) test, retaining only those with *p*_HEIDI > 0.05.

The final, robust candidates considered for colocalization analysis were selected based on a stringent cross-omics convergence criterion. This required a gene to meet the significance and HEIDI test criteria independently across all three available molecular data types for a given pain phenotype. This strategy of requiring consistent evidence from multiple, independent biological layers serves as the primary filter to minimize false-positive findings in this exploratory study. All SMR and HEIDI tests were conducted using the SMR software package (version 1.3.1) [56].

### 4.5. Colocalization Analysis for Evaluating Shared Genetic Signals Between Traits

In order to further investigate potential causal interpretations derived from SMR analyses, statistical colocalization was conducted utilizing the “coloc” R package (version 5.2.3) [57]. This methodology determines the likelihood that the associations observed between mitochondrial quantitative trait loci (QTLs) and neuralgia phenotypes arise from a common causal variant, as opposed to being attributable to distinct variants or other confounding factors. The “coloc” approach employs locus-specific summary statistics alongside an Approximate Bayes Factor (ABF) framework to calculate posterior probabilities (PP) for five mutually exclusive hypotheses: H0 (no association), H1 (association with mitochondrial trait only), H2 (association with neuralgia only), H3 (distinct causal variants for each trait), and H4 (a single shared causal variant for both traits).

Genomic loci were delineated as ±500 kb for mitochondrial QTLs, and ±1000 kb for expression QTL (eQTL) and protein QTL (pQTL) data [58,59,60]. Default prior probabilities were used for colocalization analysis, with 10^−4^ for association with the mitochondrial trait only, 10^−4^ for association with the neuralgia phenotype only, and 10^−5^ for a shared association. In accordance with established conventions, robust evidence for colocalization was defined as PP.H4.abf > 0.7.

### 4.6. Enrichment Analysis

To clarify the biological relevance of the identified mitochondrial genes, gene set enrichment analyses were performed using the clusterProfiler package (version 4.6.2) in R. Functional annotations were examined against gene ontology (GO) domains (Biological Process, Molecular Function, and Cellular Component) and the Kyoto Encyclopedia of Genes and Genomes (KEGG) pathway database. Significant enrichment was determined by a Benjamini–Hochberg adjusted *p*-value (p.adjust) < 0.05 and a minimum of two genes from the input list annotated to the term (gene count ≥ 2).

For visualization, the relationships between significantly enriched GO terms were presented as a network map (enrichment map), generated using the emapplot( ) function. In this visualization, GO terms are represented as nodes, and an edge is created between two nodes if they share common genes. The node size is proportional to the number of genes within the GO term, and the node color indicates the degree of statistical significance (p.adjust). This approach not only shows the significant terms but also illustrates the functional relationships between them.

## 5. Conclusions

Despite its limitations, this study offers valuable insights by systematically applying a rigorous multi-omics SMR framework to investigate mitochondrial genes across multiple common pain phenotypes—a relatively neglected area. Our approach identified 18 candidate genes with multi-layer regulatory links. Among these, we characterized a robust protective association for the gene GATM, which is central to creatine biosynthesis, with common musculoskeletal pain. Moreover, our expanded colocalization analysis helped to characterize the complex genetic architecture of these associations, identifying a high-confidence candidate for hip pain through a shared causal variant at a methylation QTL (mQTL) of the MCL1 gene, and revealing other loci likely driven by linkage disequilibrium. These findings underscore the intricate relationship between mitochondrial function and pain susceptibility, highlighting specific genes—notably GATM and MCL1—that warrant prioritized follow-up to validate their potential causal roles and therapeutic relevance.

## Figures and Tables

**Table 1:** Mitochondrial-related genes identified by multi-omics SMR analyses across seven neuralgia phenotypes.

Neuralgia Phenotype	Significant mQTL Associations (CpG Sites) ^1^	Significant mQTL Associations (Unique Genes) ^1^	Significant eQTL Associations (Unique Genes) ^1^	Significant pQTL Associations (Unique Genes) ^1^	Integrated Genes (Significant Across m/e/pQTL) ^2^	Evidence of Strong Colocalization ^3^
Headache	1797	368	82	10	ETFA, GRHPR, MMAB	No
Facial pain	646	243	45	8	FASN, SPHK2	No
Neck or shoulder pain	1275	361	75	10	GATM, GSTZ1, HIBCH, PRDX6	No
Back pain	1340	373	78	8	ACSF2, ECHS1, GATM	No
Stomach or abdominal pain	993	318	63	11	NME4, RMDN1, QDPR	No
Hip pain	1014	317	67	7	FAHD1, MCL1	YES (MCL1 mQTL)
Knee pain	1421	394	93	9	DBI, DCXR, GATM	No

Summary statistics of summary-data-based Mendelian randomization (SMR) analyses linking mitochondrial-related molecular features (methylation QTLs-mQTLs, expression QTLs-eQTLs, protein QTLs-pQTLs) to seven neuralgia phenotypes. ^1^ Columns indicate the number of CpG sites or unique genes showing a significant summary-data-based Mendelian randomization (SMR) association (*p*_SMR < 0.05) that also passed the HEIDI test (*p*_HEIDI > 0.05). ^2^ Genes listed met the SMR significance criteria (*p*_SMR < 0.05, *p*_HEIDI > 0.05) across all three available omic levels (mQTL, eQTL, and pQTL) for the respective phenotype. ^3^ Indicates whether strong evidence for colocalization (PP.H4.abf > 0.7) was found between the integrated QTL signals and the pain phenotype GWAS signal for the genes listed in the previous column. One association, between a methylation QTL for MCL1 and hip pain, met this threshold in our expanded analysis (PP.H4 = 0.962). ETFA: Electron transfer flavoprotein subunit alpha; GRHPR: Glyoxylate reductase/hydroxypyruvate reductase; MMAB: Metabolism of cobalamin associated B; FASN: Fatty acid synthase; SPHK2: Sphingosine kinase 2; GATM: Glycine amidinotransferase; GSTZ1: Glutathione S-transferase zeta 1; HIBCH: 3-Hydroxyisobutyryl-CoA hydrolase; PRDX6: Peroxiredoxin 6; ACSF2: Acyl-CoA synthetase family member 2; ECHS1: Enoyl-CoA hydratase, short chain 1; NME4: NME/NM23 nucleoside diphosphate kinase 4; RMDN1: Regulator of microtubule dynamics 1; QDPR: Quinoid dihydropteridine reductase, FAHD1: Fumarylacetoacetate hydrolase domain containing 1; MCL1: MCL1 apoptosis regulator; DBI: Diazepam binding inhibitor; DCXR: Dicarbonyl and L-xylulose reductase.

## Data Availability

The datasets used and/or analyzed during the current study are available from the corresponding author on reasonable request.

## Supplementary Materials

The following supporting information can be downloaded at https://www.mdpi.com/article/10.3390/ijms26178690/s1.

## Abbreviations

mtDNA	Mitochondrial DNA
SMR	Summary-data-based Mendelian randomization
GWAS	Genome-wide association study
mQTL	DNA methylation quantitative trait loci
eQTL	Gene expression quantitative trait loci
pQTL	Protein quantitative trait loci
LD	Linkage disequilibrium
SNP	Single nucleotide polymorphism
FDR	False discovery rate
HEIDI	Heterogeneity in dependent instruments
GO	Gene ontology
BP	Biological process
MF	Molecular function
CC	Cellular component
KEGG	Kyoto Encyclopedia of Genes and Genomes
ETFA	Electron transfer flavoprotein subunit alpha
GRHPR	Glyoxylate reductase/hydroxypyruvate reductase
MMAB	Metabolism of cobalamin associated B
FASN	Fatty acid synthase
SPHK2	Sphingosine kinase 2
GATM	Glycine amidinotransferase
GSTZ1	Glutathione S-transferase zeta 1
HIBCH	3-Hydroxyisobutyryl-CoA hydrolase
PRDX6	Peroxiredoxin 6
ACSF2	Acyl-CoA synthetase family member 2
ECHS1	Enoyl-CoA hydratase, short chain 1
NME4	NME/NM23 nucleoside diphosphate kinase 4
RMDN1	Regulator of microtubule dynamics 1
QDPR	Quinoid dihydropteridine reductase
FAHD1	Fumarylacetoacetate hydrolase domain containing 1
MCL1	MCL1 apoptosis regulator
DBI	Diazepam binding inhibitor
DCXR	Dicarbonyl and L-xylulose reductase

## References

[1] Awad-Igbaria Y., Ben-Menashe A., Sakas R., Edelman D., Fishboom T., Shamir A., Soustiel J.F., Palzur E. (2025). Novel Insight into TRPV1-Induced Mitochondrial Dysfunction in Neuropathic Pain. Brain J. Neurol..

[2] Espinoza N., Papadopoulos V. (2025). Role of Mitochondrial Dysfunction in Neuropathy. Int. J. Mol. Sci..

[3] Zajączkowska R., Kocot-Kępska M., Leppert W., Wrzosek A., Mika J., Wordliczek J. (2019). Mechanisms of Chemotherapy-Induced Peripheral Neuropathy. Int. J. Mol. Sci..

[4] Taschler B., Smith S.M., Nichols T.E. (2022). Causal Inference on Neuroimaging Data with Mendelian Randomisation. Neuroimage.

[5] Fang Q., Fan H., Li Q., Zhang M., Zhou Z., Du J., Huang J. (2025). Multi-Omic Insight Into the Molecular Networks in the Pathogenesis of Coronary Artery Disease. J. Am. Heart Assoc..

[6] Wang M., Xu S. (2019). Statistical Power in Genome-Wide Association Studies and Quantitative Trait Locus Mapping. Heredity.

[7] Rath S., Sharma R., Gupta R., Ast T., Chan C., Durham T.J., Goodman R.P., Grabarek Z., Haas M.E., Hung W.H.W. (2021). MitoCarta3.0: An Updated Mitochondrial Proteome Now with Sub-Organelle Localization and Pathway Annotations. Nucleic Acids Res..

[8] McRae A.F., Marioni R.E., Shah S., Yang J., Powell J.E., Harris S.E., Gibson J., Henders A.K., Bowdler L., Painter J.N. (2018). Identification of 55,000 Replicated DNA Methylation QTL. Sci. Rep..

[9] Võsa U., Claringbould A., Westra H.-J., Bonder M.J., Deelen P., Zeng B., Kirsten H., Saha A., Kreuzhuber R., Yazar S. (2021). Large-Scale Cis- and Trans-eQTL Analyses Identify Thousands of Genetic Loci and Polygenic Scores That Regulate Blood Gene Expression. Nat. Genet..

[10] Ferkingstad E., Sulem P., Atlason B.A., Sveinbjornsson G., Magnusson M.I., Styrmisdottir E.L., Gunnarsdottir K., Helgason A., Oddsson A., Halldorsson B.V. (2021). Large-Scale Integration of the Plasma Proteome with Genetics and Disease. Nat. Genet..

[11] Pinti M.V., Fink G.K., Hathaway Q.A., Durr A.J., Kunovac A., Hollander J.M. (2019). Mitochondrial Dysfunction in Type 2 Diabetes Mellitus: An Organ-Based Analysis. Am. J. Physiol.-Endocrinol. Metab..

[12] Haodong Z., Baoping C., Jiongjiong C., Jia C., Hui L., Yu W., Bocheng D. (2025). Dissecting the Genetic Etiology of Intestinal Obstruction: Mendelian Randomization Identifies Potential Therapeutic Targets. Preprint MedRxiv..

[13] Zuber V., Grinberg N.F., Gill D., Manipur I., Slob E.A.W., Patel A., Wallace C., Burgess S. (2022). Combining Evidence from Mendelian Randomization and Colocalization: Review and Comparison of Approaches. Am. J. Hum. Genet..

[14] Giambartolomei C., Liu J.Z., Zhang W., Hauberg M., Shi H., Boocock J., Pickrell J., E Jaffe A., Pasaniuc B., The CommonMind Consortium (2018). CommonMind Consortium; Pasaniuc, B.; et al. A Bayesian Framework for Multiple Trait Colocalization from Summary Association Statistics. Bioinforma. Oxf. Engl..

[15] Hemani G., Bowden J., Davey Smith G. (2018). Evaluating the Potential Role of Pleiotropy in Mendelian Randomization Studies. Hum. Mol. Genet..

[16] Cao X., Sun H., Feng R., Mazumder R., Najar C.F.B.A., Li Y.I., de Jager P.L., Bennett D., Consortium T.A.D.F.G., Dey K.K. (2025). Integrative Multi-Omics QTL Colocalization Maps Regulatory Architecture in Aging Human Brain. Preprint MedRxiv..

[17] Wallace C. (2021). A More Accurate Method for Colocalisation Analysis Allowing for Multiple Causal Variants. PLoS Genet..

[18] Lonsdale J., Thomas J., Salvatore M., Phillips R., Lo E., Shad S., Hasz R., Walters G., Garcia F., Young N. (2013). The Genotype-Tissue Expression (GTEx) Project. Nat. Genet..

[19] Wallimann T., Tokarska-Schlattner M., Schlattner U. (2011). The Creatine Kinase System and Pleiotropic Effects of Creatine. Amino Acids.

[20] Kreider R.B., Stout J.R. (2021). Creatine in Health and Disease. Nutrients.

[21] Amiri E., Sheikholeslami-Vatani D. (2023). The Role of Resistance Training and Creatine Supplementation on Oxidative Stress, Antioxidant Defense, Muscle Strength, and Quality of Life in Older Adults. Front. Public Health.

[22] Munoz H.I., Gonzales E.B., Sumien N. (2018). Effects of Creatine Supplementation on Nociception in Young Male and Female Mice. Pharmacol. Rep. PR.

[23] Meftahi G.H., Hatef B., Pirzad Jahromi G. (2023). Creatine Activity as a Neuromodulator in the Central Nervous System. Arch. Razi Inst..

[24] Wu K., Shieh J.-S., Qin L., Guo J.J. (2024). Mitochondrial Mechanisms in the Pathogenesis of Chronic Inflammatory Musculoskeletal Disorders. Cell Biosci..

[25] Morciano G., Giorgi C., Balestra D., Marchi S., Perrone D., Pinotti M., Pinton P. (2016). Mcl-1 Involvement in Mitochondrial Dynamics Is Associated with Apoptotic Cell Death. Mol. Biol. Cell.

[26] Sancho M., Leiva D., Lucendo E., Orzáez M. (2022). Understanding MCL1: From Cellular Function and Regulation to Pharmacological Inhibition. Febs J..

[27] Wajner M., Coelho J.C. (1997). Neurological Dysfunction in Methylmalonic Acidaemia Is Probably Related to the Inhibitory Effect of Methylmalonate on Brain Energy Production. J. Inherit. Metab. Dis..

[28] Sun A., Ni Y., Li X., Zhuang X., Liu Y., Liu X., Chen S. (2014). Urinary Methylmalonic Acid as an Indicator of Early Vitamin B12 Deficiency and Its Role in Polyneuropathy in Type 2 Diabetes. J. Diabetes Res..

[29] Togha M., Jahromi S.R., Ghorbani Z., Martami F., Seifishahpar M., Seifishahpar M. (2019). Serum Vitamin B12 and Methylmalonic Acid Status in Migraineurs: A Case-Control Study. Headache.

[30] García-González A., Gaxiola-Robles R., Zenteno-Savín T. (2015). Oxidative Stress in Patients with Rheumatoid Arthritis. Rev. Investig. Clin. Organo Hosp. Enfermedades Nutr..

[31] Sui B.-D., Xu T.-Q., Liu J.-W., Wei W., Zheng C.-X., Guo B.-L., Wang Y.-Y., Yang Y.-L. (2013). Understanding the Role of Mitochondria in the Pathogenesis of Chronic Pain. Postgrad. Med. J..

[32] Yorns W.R., Hardison H.H. (2013). Mitochondrial Dysfunction in Migraine. Semin. Pediatr. Neurol..

[33] Cramer S.D., Ferree P.M., Lin K., Milliner D.S., Holmes R.P. (1999). The Gene Encoding Hydroxypyruvate Reductase (GRHPR) Is Mutated in Patients with Primary Hyperoxaluria Type II. Hum. Mol. Genet..

[34] Salles J., Sargueil F., Knoll-Gellida A., Witters L.A., Shy M., Jiang H., Cassagne C., Garbay B. (2002). Fatty Acid Synthase Expression during Peripheral Nervous System Myelination. Mol. Brain Res..

[35] Squillace S., Spiegel S., Salvemini D. (2020). Targeting the Sphingosine-1-Phosphate Axis for Developing Non-Narcotic Pain Therapeutics. Trends Pharmacol. Sci..

[36] Carrasco C., Naziroǧlu M., Rodríguez A.B., Pariente J.A. (2018). Neuropathic Pain: Delving into the Oxidative Origin and the Possible Implication of Transient Receptor Potential Channels. Front. Physiol..

[37] Ferdinandusse S., Waterham H.R., Heales S.J.R., Brown G.K., Hargreaves I.P., Taanman J.-W., Gunny R., Abulhoul L., Wanders R.J.A., Clayton P.T. (2013). HIBCH Mutations Can Cause Leigh-like Disease with Combined Deficiency of Multiple Mitochondrial Respiratory Chain Enzymes and Pyruvate Dehydrogenase. Orphanet J. Rare Dis..

[38] Asuni A.A., Guridi M., Sanchez S., Sadowski M.J. (2015). Antioxidant Peroxiredoxin 6 Protein Rescues Toxicity Due to Oxidative Stress and Cellular Hypoxia in Vitro, and Attenuates Prion-Related Pathology in Vivo. Neurochem. Int..

[39] Wagner K., Lee K.S.S., Yang J., Hammock B.D. (2017). Epoxy Fatty Acids Mediate Analgesia in Murine Diabetic Neuropathy. Eur. J. Pain Lond. Engl..

[40] Wang B., Qin Y., Bao Y., Chen S., Zheng J., Lin S., Zheng K., Duan S. (2025). Deficiency in the Conserved ECHS1 Gene Causes Leigh Syndrome by Impairing Mitochondrial Respiration Efficiency and Suppressing ADRB2-PKA Signaling. Biochim. Biophys. Acta BBA-Mol. Basis Dis..

[41] Lacombe M.-L., Tokarska-Schlattner M., Boissan M., Schlattner U. (2018). The Mitochondrial Nucleoside Diphosphate Kinase (NDPK-D/NME4), a Moonlighting Protein for Cell Homeostasis. Lab. Invest..

[42] Oishi K., Okano H., Sawa H. (2007). RMD-1, a Novel Microtubule-Associated Protein, Functions in Chromosome Segregation in Caenorhabditis Elegans. J. Cell Biol..

[43] Latremoliere A., Costigan M. (2011). GCH1, BH4 and Pain. Curr. Pharm. Biotechnol..

[44] Lim T.K.Y., Rone M.B., Lee S., Antel J.P., Zhang J. (2015). Mitochondrial and Bioenergetic Dysfunction in Trauma-Induced Painful Peripheral Neuropathy. Mol. Pain.

[45] Enna S.J., McCarson K.E. (2006). The Role of GABA in the Mediation and Perception of Pain. Adv. Pharmacol. San Diego Calif..

[46] Wada R., Yagihashi S. (2005). Role of Advanced Glycation End Products and Their Receptors in Development of Diabetic Neuropathy. Ann. N. Y. Acad. Sci..

[47] Davies N.M., Holmes M.V., Davey Smith G. (2018). Reading Mendelian Randomisation Studies: A Guide, Glossary, and Checklist for Clinicians. BMJ.

[48] Chepelev I., Harley I.T.W., Harley J.B. (2023). Modeling of Horizontal Pleiotropy Identifies Possible Causal Gene Expression in Systemic Lupus Erythematosus. Front. Lupus.

[49] Relton C.L., Smith G.D. (2015). Mendelian Randomization: Applications and Limitations in Epigenetic Studies. Epigenomics.

[50] Arvanitis M., Tayeb K., Strober B.J., Battle A. (2022). Redefining Tissue Specificity of Genetic Regulation of Gene Expression in the Presence of Allelic Heterogeneity. Am. J. Hum. Genet..

[51] Sonawane A.R., Platig J., Fagny M., Chen C.-Y., Paulson J.N., Lopes-Ramos C.M., DeMeo D.L., Quackenbush J., Glass K., Kuijjer M.L. (2017). Understanding Tissue-Specific Gene Regulation. Cell Rep..

[52] Purcell S., Neale B., Todd-Brown K., Thomas L., Ferreira M.A.R., Bender D., Maller J., Sklar P., de Bakker P.I.W., Daly M.J. (2007). PLINK: A Tool Set for Whole-Genome Association and Population-Based Linkage Analyses. Am. J. Hum. Genet..

[53] Skrivankova V.W., Richmond R.C., Woolf B.A.R., Davies N.M., Swanson S.A., VanderWeele T.J., Timpson N.J., Higgins J.P.T., Dimou N., Langenberg C. (2021). Strengthening the Reporting of Observational Studies in Epidemiology Using Mendelian Randomisation (STROBE-MR): Explanation and Elaboration. BMJ.

[54] Elsworth B., Lyon M., Alexander T., Liu Y., Matthews P., Hallett J., Bates P., Palmer T., Haberland V., Smith G.D. (2020). The MRC IEU OpenGWAS Data Infrastructure. Preprint bioRxiv.

[55] Zhu Z., Zhang F., Hu H., Bakshi A., Robinson M.R., Powell J.E., Montgomery G.W., Goddard M.E., Wray N.R., Visscher P.M. (2016). Integration of Summary Data from GWAS and eQTL Studies Predicts Complex Trait Gene Targets. Nat. Genet..

[56] Sun Z., Yun Z., Lin J., Sun X., Wang Q., Duan J., Li C., Zhang X., Xu S., Wang Z. (2024). Comprehensive Mendelian Randomization Analysis of Plasma Proteomics to Identify New Therapeutic Targets for the Treatment of Coronary Heart Disease and Myocardial Infarction. J. Transl. Med..

[57] Giambartolomei C., Vukcevic D., Schadt E.E., Franke L., Hingorani A.D., Wallace C., Plagnol V. (2014). Bayesian Test for Colocalisation between Pairs of Genetic Association Studies Using Summary Statistics. PLoS Genet..

[58] (2017). GTEx Consortium Genetic Effects on Gene Expression across Human Tissues. Nature.

[59] Morrow J.D., Glass K., Cho M.H., Hersh C.P., Pinto-Plata V., Celli B., Marchetti N., Criner G., Bueno R., Washko G. (2018). Human Lung DNA Methylation Quantitative Trait Loci Colocalize with Chronic Obstructive Pulmonary Disease Genome-Wide Association Loci. Am. J. Respir. Crit. Care Med..

[60] Yoshiji S., Butler-Laporte G., Lu T., Willett J.D.S., Su C.-Y., Nakanishi T., Morrison D.R., Chen Y., Liang K., Hultström M. (2023). Proteome-Wide Mendelian Randomization Implicates Nephronectin as an Actionable Mediator of the Effect of Obesity on COVID-19 Severity. Nat. Metab..

